# Correlation between early corneal edema and endothelial cell loss after phacoemulsification cataract surgery

**DOI:** 10.3389/fmed.2025.1562717

**Published:** 2025-05-29

**Authors:** Si Chen, Xiaoqing Li, Li Huang, Qin Feng, Huilong Lu, Jing Mu

**Affiliations:** ^1^Department of Ophthalmology, Jinshan Branch of Shanghai Sixth People’s Hospital, Shanghai, China; ^2^Department of Ophthalmology, Shanghai Sixth People’s Hospital Affiliated to Shanghai Jiao Tong University School of Medicine, Shanghai, China

**Keywords:** corneal edema, corneal endothelial cells, cataract, phacoemulsification, correlation

## Abstract

**Purpose:**

This study aimed to investigate the correlation between early corneal edema and loss of corneal endothelial cells after phacoemulsification.

**Methods:**

The corneal condition of each operated eye was observed using slit-lamp biomicroscopy at different time points, and the clinical score of corneal edema was determined. Central corneal thickness (CCT) and endothelial cell density (ECD) of each operative eye were measured by corneal endothelial microscopy at different time points.

**Results:**

There were 10 male (41.67%) and 14 female (58.33%) cataract patients with a mean age of aged 71.88 ± 10.14 years. The clinical score of corneal edema grade in cataract patients on the first postoperative day (1.79 ± 1.10) was significantly higher than that before surgery (0.0 ± 0.0) (*p* < 0.0001). Similarly, the CCT on first postoperative day (566.08 ± 32.73 μm) was significantly higher than that before surgery (530.71 ± 24.42 μm) (*p* < 0.0001). In addition, compared with the preoperative ECD (2,841 ± 502 cells/mm^2^), the ECD (1,993 ± 744 cells/mm^2^) 1 month after surgery (M1) decreased significantly (*p* < 0.0001), and the percentage of endothelial cell loss was 30.45 ± 20.23%. Moreover, both the corneal edema grade (Spearman’s *r* = 0.7811, *p* < 0.0001) and CCT (Spearman’s *r* = 0.7191, *p* < 0.0001) on the first day after surgery were significantly correlated with the loss of corneal endothelial cells 1 month postoperative day.

**Conclusion:**

Early corneal edema after cataract surgery is closely associated with the loss of central corneal endothelial cells. Therefore, the grade of corneal edema and CCT on the first day after cataract surgery can be used as effective indices for predicting the effects of phacoemulsification on corneal endothelial cells.

## Introduction

Cataracts are not only common blinding ocular diseases but also major global public health problems. According to statistical data, among the total number of blind people in the world, the number of cataract-related blindness accounts for 33.4% of all cases of blindness worldwide, representing more than 10 million individuals (1, 2). There are various types of cataracts, with age-related cataracts being the most common. The occurrence of age-related cataracts is closely associated with age; in other words, as age increases, so does the incidence of cataracts (20). Currently, cataract extraction combined with intraocular lens (IOL) implantation is the primary treatment for cataracts. One of the common complications of cataract surgery is corneal edema, which may progress to corneal endothelial decompensation in severe cases.

Endothelial cell dysfunction or injury may lead to changes in corneal permeability, resulting in corneal edema and even bullous keratopathy (3). Endothelial cell density (ECD) is commonly used to quantify the number of corneal endothelial cells, typically measured at the central cornea. Studies have shown that the ECD in healthy young individuals is 3109.0 ± 303.7 cells/mm^2^ and is significantly and negatively correlated with age (4). Given that corneal endothelial cells are nonregenerative, potential causes of endothelial damage should be minimized whenever possible. Previous studies have shown that cataract surgery is a common cause of corneal endothelial loss (3).

In clinical practice, experienced ophthalmologists can directly assess the severity of corneal edema by observing the state of the cornea using a slit-lamp biomicroscope (5). For example, Aggarwal et al. (6) reported grade 2 to 3 diffuse central corneal edema and stromal folds in the right eye of an elderly cataract patient after phacoemulsification, accompanied by a marked increase in corneal thickness. In addition, central corneal thickness (CCT) has been widely used to assess corneal edema (7). In this study, we investigated the correlation between corneal edema and endothelial cell injury after phacoemulsification.

## Methods

This prospective observational study was conducted at the Ophthalmology Department of the Jinshan Branch of Shanghai Sixth People’s Hospital from February 2023 to January 2024. All procedures were performed in accordance with the Declaration of Helsinki guidelines. This study was approved by the Ethics Committee of Shanghai Sixth People’s Hospital Jinshan Branch. Written informed consent was obtained from all participants prior to surgery. Consent was obtained by the operating surgeon during the preoperative consultation. Researchers were responsible for data collection and outcome assessment but did not participate in the surgical procedures or the consent process.

### Patients

All patients with cataracts were recruited from the Jinshan Branch of Shanghai Sixth People’s Hospital. We selected patients diagnosed with age-related cataracts and who underwent phacoemulsification combined with IOL implantation as study subjects. Individuals with the following conditions were excluded from the study: (1) other ocular diseases affecting vision (e.g., glaucoma, uveitis, macular edema); (2) a history of ocular surgery or trauma; (3) keratopathy (e.g., corneal scarring, corneal opacity, corneal endothelial dystrophy); and (4) systemic diseases (e.g., dementia, ankylosing spondylitis). All patients were older than 18 years of age. The same senior ophthalmologist performed phacoemulsification and IOL implantation. Surface anesthesia, a clear incision of the cornea, and a phacoemulsification surgery system (CV-9000R, NIDEK, Japan) were used, and a foldable IOL was implanted in the posterior capsule. All patients were followed up for 1 month. To avoid inter-eye correlation, only one eye per patient was selected for inclusion in the analysis.

### Grading of corneal edema

Corneal edema in each operated eye was evaluated by another senior ophthalmologist using slit-lamp microscopy (S360, Mediworks, Shanghai, China) before surgery and postoperative day 1, week 2, and month 1. According to a previously reported clinical grading system for corneal edema (5), the severity of corneal edema in each operated eye was graded on a scale of 0–4. Table 1 shows the main clinical scoring criteria: corneal transparency and changes in the corneal epithelium, stromal layer and posterior elastic layer.

**Table 1:** Clinical scoring system for corneal edema.

Clinical sign				Score
Corneal transparency	Corneal epithelium	Corneal stroma	Descemet’s membrane	
Clear	—	—	—	0
Mild opacity	Localized edema	Mild edema	—	1
Moderate opacity	Diffuse edema	And/or diffuse edema	Mild folds	2
Serious opacity	Focal microcystic edema or bullae	And/or moderate-to-high stromal thickening	Moderate folds and haze	3
Serious opacity	Diffuse bullous keratopathy	And/or moderate-to-high stromal thickening	And/or moderate folds and haze	4

### Endothelial cell and corneal thickness detection

Corneal central ECD and CCT were measured using corneal endothelial microscopy (SP-1P Topcon, Japan) before surgery and postoperative day 1, week 2, and month 1. Each measurement was repeated three times for each eye, and the average value was calculated.

### Statistical analysis

The values were expressed as mean ± standard deviation. Differences were analyzed using Student’s *t*-test and one-way ANOVA with Tukey’s multiple comparisons test. The paired Student’s *t*-test was used to compare the differences in ECD between the same individual before and1 month after surgery. When comparing data from more than two time points, one-way ANOVA with Tukey’s multiple comparisons test analysis of variance was used. Spearman’s correlation was used to analyze the correlation between corneal edema grade and endothelial cell loss. Statistical analysis was performed using GraphPad Prism 9.2.0 (GraphPad, La Jolla, CA, United States), and statistical significance was set at *p* < 0.05.

## Results

A total of 24 patients with senile cataracts were enrolled, including 10 males (41.67%) and 14 females (58.33%). The mean age was 71.88 ± 10.14 years, with 24 eyes. All eyes underwent phacoemulsification cataract surgery combined with intraocular lens implantation.

### Early postoperative corneal edema following phacoemulsification was significantly increased

**Figure 1:**
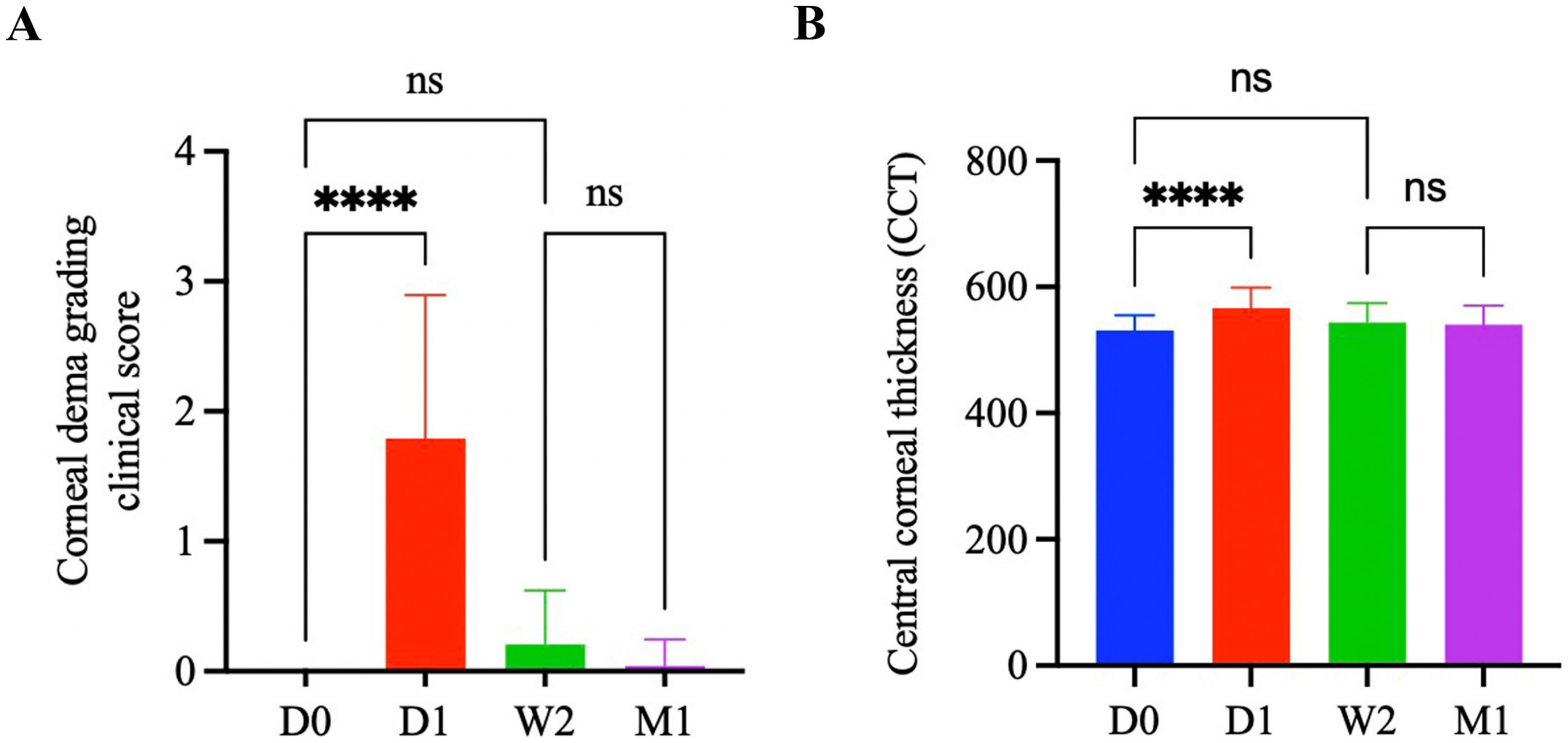
**(A)** Changes in clinical score of corneal edema at different times after phacoemulsification. The values represent the mean ± standard deviation. Significance was determined using one-way ANOVA with Tukey’s multiple comparisons test. ****p < 0.0001. ns, ns indicates no statistically significant difference (*p* > 0.05). **(B)** Changes in central corneal thickness at different times after phacoemulsification. The values represent the mean ± standard deviation. Significance was determined using one-way ANOVA with Tukey’s multiple comparisons test. ****p < 0.0001. ns, indicates no statistically significant difference (*p* > 0.05).

According to the corneal edema grading clinical scoring system (Table 1), all cataract patients were scored before surgery (D0), 1 day after surgery (D1), 2 weeks after surgery (W2) and 1 month after surgery (M1). Figure 1A shows that the clinical score of D1 (1.79 ± 1.10) was significantly higher than that of D0 (0.0 ± 0.0) (*p* < 0.0001). However, there was no significant difference between D0 and W2 (0.21 ± 0.41) or M1 (0.04 ± 0.20). Similarly, Figure 1B shows that CCT of cataract patients on D1 (566.08 ± 32.73 μm) was significantly greater than that of D0 (530.71 ± 24.42 μm) (*p* < 0.0001). There was no significant difference between D0, W2 (543.33 ± 30.98 μm) and M1 (540.33 ± 30.08 μm). Hence, considering that corneal edema basically subsided in all patients 1 month after surgery, this study considered 1 month after surgery as the time point at which the decrease in corneal endothelial cells tended to stabilize.

### The density of corneal endothelial cells decreased significantly after phacoemulsification

**Figure 2:**
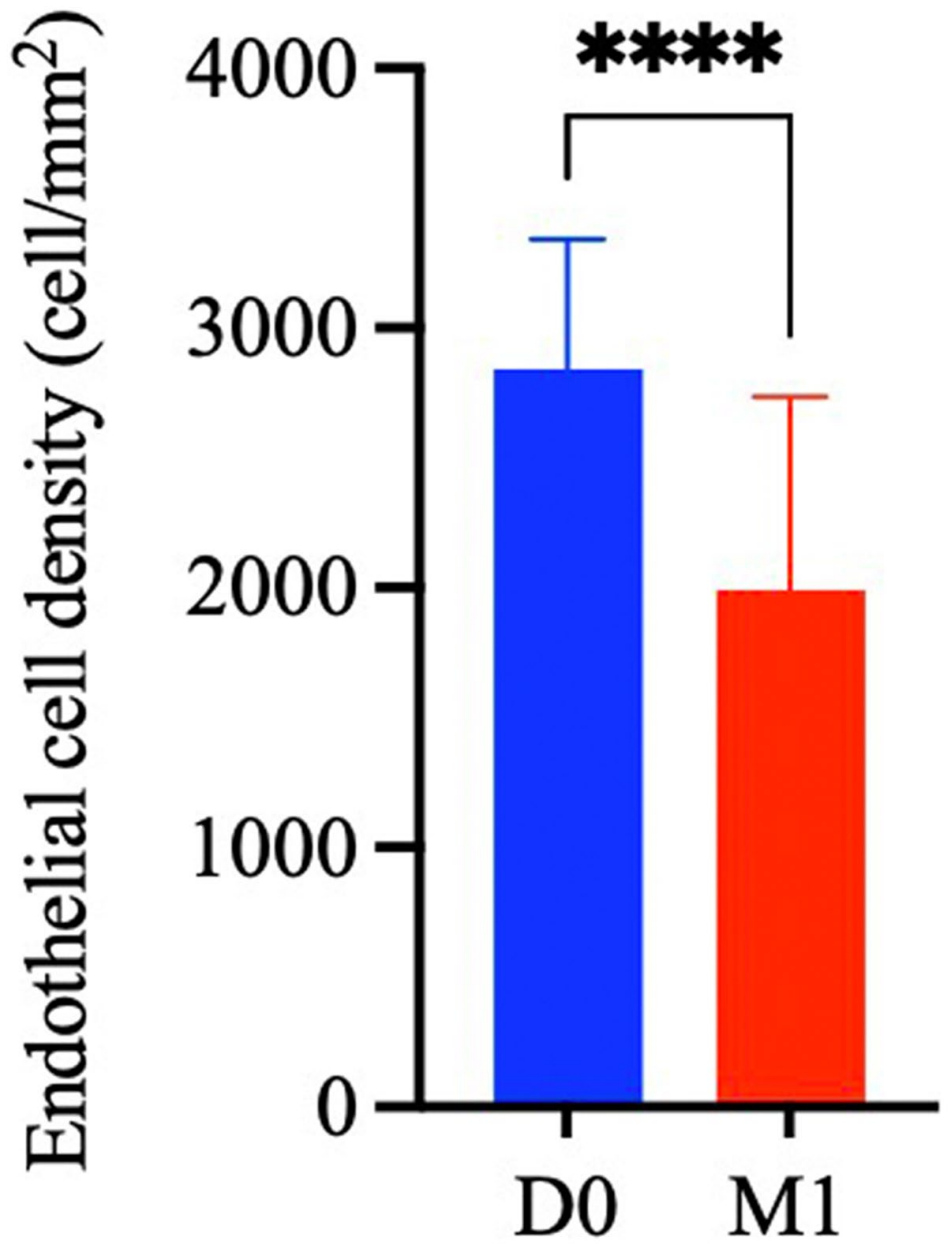
Changes in endothelial cell density before and 1 month after phacoemulsification cataract surgery. The values represent the mean ± standard deviation. Significance was determined using paired Student’s *t*-test. ****p < 0.0001.

To investigate the changes in the corneal central ECD in cataract patients after surgery, measurements were made before surgery (D0) and 1 month after surgery (M1). As shown in Figure 2, compared with the preoperative ECD (D0) (2,841 ± 502 cells/mm^2^), the ECD at 1 month post-surgery (M1) (1993 ± 744 cells/mm^2^) was significantly reduced (*p* < 0.0001), with an average endothelial cell loss rate was 30.45 ± 20.23%.

### The early corneal edema after phacoemulsification is closely related to the loss of corneal endothelial cells

**Figure 3:**
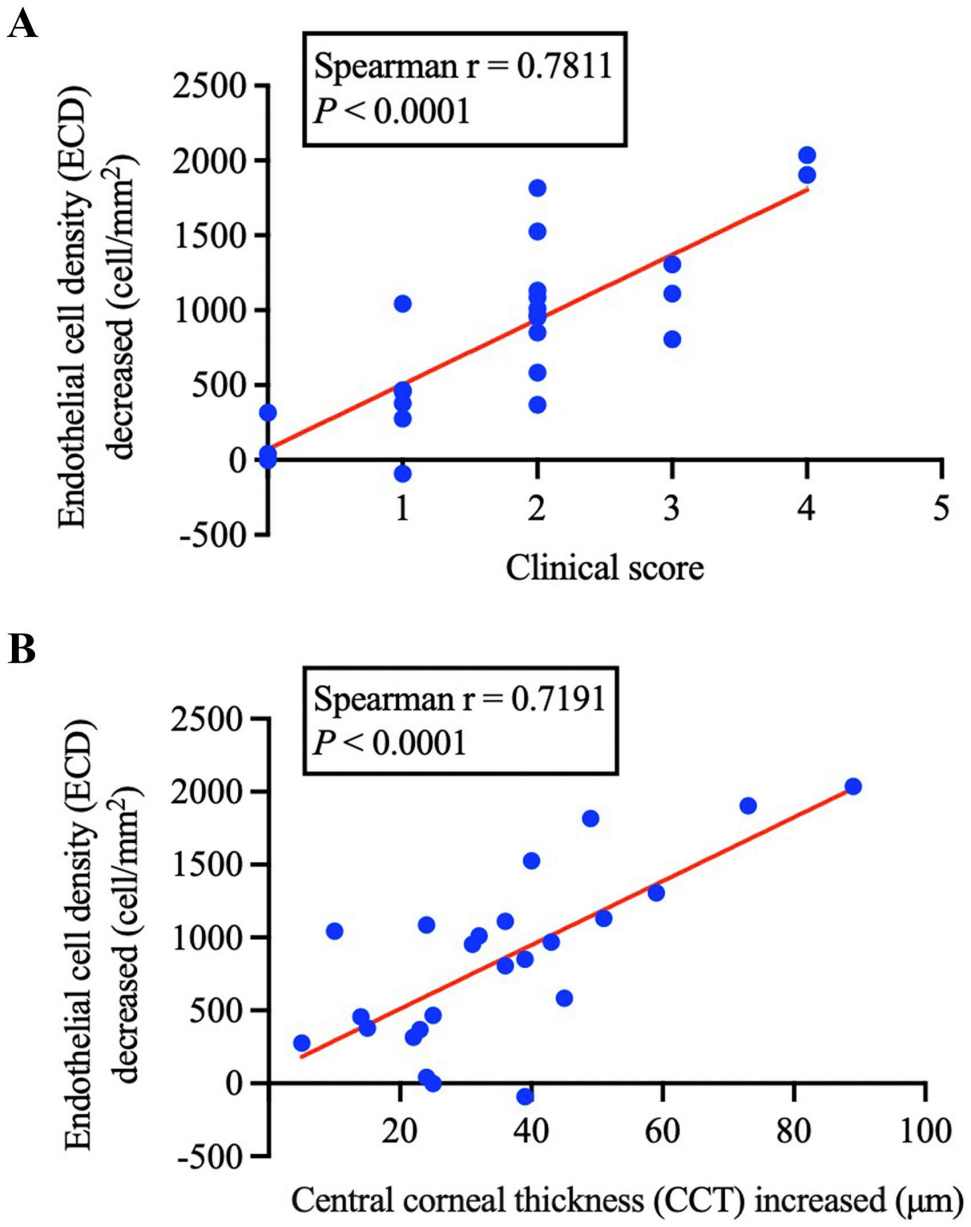
**(A)** Correlation between clinical scores of early corneal edema and endothelial cell loss after phacoemulsification. **(B)** Correlation between centre corneal thickness increase and endothelial cell loss after phacoemulsification.

To explore the correlation between early postoperative corneal edema and corneal endothelial cell loss in patients with cataract, we conducted correlation analysis. As shown in Figure 3A, the clinical score of corneal edema grade in cataract patients on the first day after surgery was strongly correlated with the loss of corneal endothelial cells 1 month after surgery (Spearman’s *r* = 0.7811, *p* < 0.0001). Consistent with these findings, the increase of CCT on the first day after surgery was strongly correlated with the loss of corneal endothelial cells 1 month after surgery (Spearman’s *r* = 0.7191, *p* < 0.0001) (Figure 3B). These results revealed that the severity of corneal edema in early postoperative cataract patients is positively correlated with the loss of corneal endothelial cells.

## Discussion

This study investigated changes in corneal edema and ECD after phacoemulsification and the correlation between them. We found that the clinical score of corneal edema and CCT increased significantly on the first day after surgery, while the central corneal ECD decreased significantly in the first month after surgery. Interestingly, the grade of corneal edema on day 1 after surgery was closely associated with decreased endothelial cell count.

In this study, we focused on the clinical grade of corneal edema on day one after cataract surgery. In previous studies, CCT was a common measure of corneal edema (8, 9). However, the relationship between the clinical grade of postoperative corneal edema and endothelial cell loss has not been reported. The clinical grade of corneal edema can be directly evaluated using slit-lamp microscopy, which is of great clinical significance in countries/regions where corneal endothelial microscopy or optical coherence tomography (OCT) is not widely available. Therefore, different methods were used to evaluate the degree of early corneal edema after phacoemulsification cataract surgery to explore the correlation between corneal edema and corneal endothelial cell loss.

This study revealed that the central corneal ECD decreased significantly 1 month after phacoemulsification. To explore the reasons for the decrease in corneal endothelial cells after cataract surgery, many scholars have conducted related studies. For example, Ungricht et al. (10, 11) reported that torsional phacoemulsification caused much less damage to corneal endothelial cells than classical longitudinal phacoemulsification and that balanced salt solution flow alone was not a major factor in endothelial cell damage or loss. Additionally, Wilkinson et al. (12) reported that the rotation of lens fragments near the anterior chamber during phacoemulsification may be one of the factors causing severe damage to the corneal endothelium. However, Kim et al. (13) reported that the recovery of corneal endothelial cells was mainly affected by cell enlargement in the early stage after cataract surgery. Hwang et al. reported that the use of mannitol before cataract surgery reduced the loss of corneal endothelial cells after surgery.

This study revealed that corneal edema on the first day after phacoemulsification was closely related to endothelial cell loss. The degree of endothelial cell injury can be predicted in advance according to the early postoperative corneal edema. The specific mechanism of endothelial cell loss after cataract surgery is still unclear, and further studies are needed, especially on the relationship between the corneal endothelium and corneal edema. Previous studies have shown that abnormal corneal endothelial cell function may lead to corneal edema (14). Close attention should be paid to severe postoperative corneal edema. Excessive loss of endothelial cells may lead to the occurrence of corneal endothelial decompensation. Cui et al. (15) reported that Fuchs endothelial corneal dystrophy, which can develop into corneal endothelial decompensation, could be linked to genes involved in cellular senescence, reactive oxygen species (ROS), the extracellular matrix (ECM), epithelial-mesenchymal transition (EMT), and immune responses and, in severe cases, may lead to bullous keratopathy and even blindness (21).

However, some comorbidities are also factors that influence the decrease in endothelial cells after cataract surgery. The effect of diabetes mellitus, especially type 2 diabetes mellitus (T2DM), on endothelial cell loss has been extensively reported in the literature. Studies have shown that patients with diabetes experience significantly greater corneal endothelial loss after phacoemulsification in patients with diabetes than in patients without diabetes (16, 17). This association between T2DM and endothelial cell loss may be attributed to diabetes-related metabolic disorders. Notably, Yu et al. (18) demonstrated that supplementation with L-alanyl-L-glutamine (Ala-Gln) in diabetic models effectively attenuated high glucose-induced oxidative stress and endothelial apoptosis, while promoting corneal edema resolution and functional recovery. Zhang et al. (19), through diabetic mouse models and *in vitro* experiments, found that diabetes increased reactive oxygen species (ROS), down-regulated antioxidant factors, nuclear factor-erythroid 2-related factor 2 (Nrf2) and NAD (P)H: quinone oxidoreductase 1 (NQO1) expression, and reduced Nrf2 nuclear translocation, thereby leading to more serious oxidative damage to corneal endothelial cells and more obvious corneal edema. Therefore, we should pay more attention to the importance of preoperative screening of endothelial function in diabetic patients, and more stricter intraoperative protective measures are needed to minimize permanent corneal endothelial injury.

## Conclusion

In summary, corneal edema on the first postoperative day following cataract surgery was strongly associated with corneal endothelial cell loss. This finding highlights the importance of corneal management before and after phacoemulsification and provides a practical reference for predicting the impact of phacoemulsification cataract surgery on corneal endothelial cells.

## Data Availability

The original contributions presented in the study are included in the article/supplementary material, further inquiries can be directed to the corresponding author.
